# Myopia Prevalence and Associated Factors IN School-Aged Children in Southern Morocco: A Cross-Sectional Study

**DOI:** 10.22599/bioj.400

**Published:** 2025-08-25

**Authors:** Elhassane Benhim, Farida Bentayeb, Abderrahim Dahbi, R’hma Adhiri

**Affiliations:** 1 Laboratory of Engineering and Materials, Faculty of Science Ben M’sick, Hassan II University of Casablanca, B.P.7955, Casablanca, Morocco; 2 Laboratory of Electronic Systems, Mechanical and Energy Information Processing, Faculty of Science, Ibn Tofail University, Morocco

**Keywords:** myopia, vision, school-aged children, Morocco

## Abstract

Myopia is one of the leading causes of visual impairment worldwide, typically beginning during the school-age years. Several factors contribute to its development, including environmental influences, excessive use of digital devices, and limited outdoor activities. The objective of this study was to assess the prevalence of myopia and associated factors among school-aged children and adolescents in rural areas of southern Morocco. A cross-sectional study was conducted between November 2022 and January 2023, involving 342 participants, with a majority of boys (54.4%) and a mean age of 13 years. Data were collected using a questionnaire that gathered sociodemographic and vision-related information, followed by a vision test to determine myopia status using cycloplegic autorefraction. The results revealed a relatively low prevalence of myopia (11%, 95% CI: 7.8%–14.4%). Multivariate logistic regression showed that a screen-watching distance less than 35 cm was significantly associated with an increased risk of myopia (OR = 1.89; 95% CI: 1.02–3.47; p = 0.042). The majority of participants (98%) reported spending at least two hours outdoors daily, and the average daily use of digital devices was 30 minutes. Interestingly, the majority of participants (72%) maintained a screen viewing distance of ≥35 cm, which was found to be associated with myopia (p = 0.04). This may reflect a behavioral adaptation rather than a direct causal relationship. Overall, the study suggests that the low prevalence of myopia in this population may be attributed to high exposure to daylight and limited use of digital devices. These findings underscore the potential protective role of outdoor activities and highlight the need for further research to better understand the factors influencing myopia development in rural populations.

## Introduction

According to the First World Report on Vision from the World Health Organization (WHO), myopia is one of the main eye conditions inducing vision impairment worldwide and evidence shows an increase in myopia worldwide (World Health Organization, 2019).

Indeed, in 2000, an estimated 1.4 billion individuals had myopia, and projections suggest that by 2050, it will rise to 4.8 billion (Holden *et al.*, 2016). This global increase in myopia prevalence has implications for service planning, including the management and prevention of ocular complications linked to myopia, ultimately aiming to address vision loss among nearly 1 billion individuals affected by severe myopia (Holden *et al.*, 2016). Thus, the escalating prevalence of myopia poses a significant public health challenge worldwide (Grzybowski *et al.*, 2020; Morgan, 2016; Tariq *et al.*, 2023). Myopia can onset at any point in life, though it commonly begins during the school-age years in most cases (Pärssinen, Kauppinen and Viljanen, 2014). However, there is particular concern for children, as they are among the most vulnerable. Some factors are known to help prevent myopia or control its progression, such as limiting screen time in children and encouraging outdoor activities (Jones *et al.*, 2007). A study, conducted by Wang et al. aimed at investigating and evaluating the impact of lockdown showed that increased exposure to digital screens contributes to the progression of myopia in children and adolescents in Chongqing China during the COVID-19 pandemic (Wang *et al.*, 2021).

Nowadays, children devote substantial time to activities like reading, studying, or more recently, using computers and smartphones, which negatively impact the progression of myopia. Children spend more time each day on indoor activities requiring proximity compared to time spent outdoors (McCrann *et al*., 2018). Additionally, the primary identified risk factors for myopia include lack of visual education, excessive screen time, prolonged near-screen viewing, and limited outdoor activities (Morgan *et al*., 2018). This refers to the absence of awareness or education about eye health, such as the importance of limiting screen time and encouraging outdoor activities to prevent vision problems like myopia.

Concerns also arise for children with early-onset myopia, as they face prolonged disease duration, increased myopia progression, and heightened risks of developing severe myopia, including myopic macular degeneration.

Additionally, another significant factor is the higher prevalence of myopia among children residing in urban areas, as they have a 2.6 times greater likelihood of developing myopia compared to children living in rural areas (Jones *et al.*, 2007; Rudnicka *et al.*, 2016). It is well-documented in the literature that adolescents affected by myopia in urban areas spend significantly more time engaged with digital screens compared to their non-myopic counterparts from rural settings (McCrann *et al*., 2018).

The increasing prevalence of myopia poses a significant global public health challenge in African countries like Morocco (Ovenseri-Ogbomo *et al.*, 2022). Just a few years ago, the prevalence of myopia was relatively low among school-aged children, but this phenomenon has rapidly escalated worldwide (Anera *et al.*, 2009; Samya Korziti *et al.*, 2023). However, there are still few studies available on the status of myopia in Morocco, despite a growing awareness of the issue since the COVID-19 pandemic (Belkhadir and Cherkaoui, 2020).

In this regard, the objective of our study was to assess the prevalence of myopia and associated factors among school-aged children and adolescents in Southern Morocco.

## Methods

### Study design and population

This cross-sectional study was conducted between November 2022 and January 2023 among school-aged children and adolescents from two rural cities in Southern Morocco, Bleida, and Zagora.

For participant selection, a non-probabilistic convenience sample was used. The sample size (n) was determined using the following formula:


\[{\rm n}=\frac{{\rm Z}^{2} \cdot {\rm P}(1-P)}{E^2}\]

where P represents the estimated prevalence of myopia (50%), given the lack of previous studies in Morocco on this specific research topic, E is the margin of error set at 5%, and Z corresponds to a 95% confidence level (Z = 1.96)., this approach yielded a required sample size of 382 participants. After data cleaning, 40 questionnaires with incomplete data were excluded, resulting in a final sample size of 342 participants.

Participants’ ages were verified using official documents and parental statements.

The inclusion criteria for the study were: school-aged children and adolescents (ages 6–18 years) residing in the rural areas of Southern Morocco (Zagora and Bleida), and participants who provided informed consent (from themselves or their legal guardians) for cycloplegic eye examinations.

The exclusion criteria were children with a history of eye surgeries, systemic diseases, or neurological conditions that could affect visual acuity, those using medications that might interfere with eye health or refractive status, and children whose parents did not consent to the Cycloplegic eye examinations were conducted. Additionally, individuals physically unable to provide all data were also excluded from the study. Upon consenting to participate, the participants interacted with the principal investigator, a certified optician. Data collection was conducted in two phases and lasted approximately 15 minutes per participant, ensuring minimal disruption to their daily activities while maximizing compliance. The equipment used was an autorefractor (Huvitz HRK-1, 2019) in dim lighting to prevent involuntary accommodation. Cycloplegia was induced using Tropicamide 1% (Montgomery and Macewan, 1989), administered with two drops per eye, five minutes apart. Vision testing was conducted 30 minutes later, and visual acuity (VA) was assessed after cycloplegia, 30 minutes following the second instillation of Tropicamide 1%, to minimize the influence of accommodation, particularly in children. While this approach differs from standard clinical practice, where VA is typically measured before cycloplegia, the standardized measurement conditions at a distance of 6 meters and controlled lighting minimized the impact of pupil dilation and loss of accommodation on the far-vision results. Both monocular and binocular visual acuity were recorded in decimal format, with an inter-examiner verification to ensure measurement reliability.

The questionnaire, consisting of two sections, was administered by an interviewer. The first section gathered socio-demographic information, while the second focused on vision-related habits (e.g., screen time, viewing distance, outdoor exposure). The questionnaire was developed by our team based on the practical experience of vision professionals working with children. It was not adapted from any specific validated studies but rather designed to meet the study’s needs. It was first written in French, then translated into Moroccan Arabic by two bilingual professionals. The translation was reviewed by three optometrists to ensure that the questions were clear and relevant to the local context. Parents provided answers for children under 6 years old. The questionnaire was administered orally in Moroccan Arabic, with additional explanations given as needed to ensure accurate responses. The investigator documented all responses.

Reading distance was measured before administering Tropicamide by providing a text for participants to read, and the adopted distance was recorded. Additionally, the questionnaire collected information on extracurricular habits such as sun exposure and after-school reading time.

To assess myopia status, autorefraction was performed separately for both eyes under cycloplegic conditions. Myopia was defined as a spherical equivalent of less than –0.5 dioptres, astigmats, and hypermetropes. For analysis, we chose to use only the right eye, as measurements from both eyes were highly similar, allowing for a simplified approach without compromising accuracy.

### Data analysis

The information was entered into Excel for computerization and later analyzed using the Statistical Package for the Social Science (SPSS) software, version 16. Mean values, along with their respective standard deviations (SD), were used to represent quantitative variables. Numbers and percentages were used for qualitative variables. Comparisons of percentages were conducted using Fisher’s exact test, and comparisons of means were done using the Student’s t-test. The significance level was set at 0.05.

In addition, a multivariate analysis (logistic regression) was performed to identify independent risk factors for myopia, controlling for potential confounders. This analysis allowed us to determine variables independently associated with myopia, accounting for interactions between variables.

### Ethical aspects

This research was conducted following scientific and ethical principles and was submitted to, and approved by, the Biomedical Research Ethics Committee (BREC), Faculty of Medicine and Pharmacy, Mohammed Premier University of Oujda, under reference number 51/2023. All participants were informed about the primary objective of the study and provided voluntary, informed consent. Additionally, the confidentiality of all data was strictly maintained throughout the study. While no financial compensation was provided, all participants received a visual acuity examination as part of their participation in the study.

## Results

### Sociodemographic characteristics of participants

Three hundred forty-two subjects participated in this study with a majority of boys (54.4%). The participants’ mean age was 13 years (SD:2.22 years). Mainly, they studied at the secondary school (70%) and came from Bleida city (64%). The sociodemographic characteristics of participants are presented in Table 1.

**Table 1:** Sociodemographic characteristics of participants.


	CHARACTERISTICS	N = 342

**Age**		13.0 (11.0, 14.0)

**Gender**	Boys	186 (54.4%)

	Girls	156 (45.6%)

**Study levels**	Nursery school	7 (2.1%)

	Primary school	88 (26%)

	Secondary school	236 (70%)

	High school	4 (1.2%)

	University	3 (0.9%)

**Cities**	Bleida	218 (64%)

	Zagora	124 (36%)


Statistics presented: median (IQR); n (%Information related to the Refractive error of participants).

The majority of participants (89%) were non-myopic, including emmetropes, astigmats (whose spherical equivalent was greater than –0.50), and hypermetropes (spherical equivalent greater than +0.5) The prevalence of myopia was 11% (95% CI = 7.8%–14.4%).

Most of participants reported having no parental myopia (93%), and their mean after-school study time was 60 minutes per day. Moreover, they declared using digital devices for 30 minutes per day (mean time), with a screen-watching distance was mainly greater than or equal to 35 centimeters (72%). Furthermore, almost all participants reported exposure to the sun every day for a length of two hours or more (98%). Information related to the participants’ vision is presented in Table 2. No significant associations were observed between age (p = 0.98), study levels (p = 0.67), after-school study time (p = 0.46), screen-watching time (p = 0.6), exposure to daylight (p = 0.44), parental myopia (p = 0.36) and the myopia status of our participants. However, there was non-significant association with screen-watching distance (p = 0.1012).

**Table 2:** Information related to the refractive error and characteristics of participants’.


CHARACTERISTICS	TOTAL (N = 342)	MYOPIA – NO (N = 304)	MYOPIA – YES (N = 38)	P–VALUE

**Parental myopia (at least one parent)**				0.3692

No	318 (93.0%)	284 (93.4%)	34 (89.5%)	

Yes	24 (7.0%)	20 (6.6%)	4 (10.5%)	

**Age (years)**				0.9841

Mean (SD)	–	12.7 (3.2)	12.7 (3.6)	

Range	–	2.0–29.0	5.0–23.0	

**Study level**				–

Nursery school	6 (1.8%)	5.0 (1.7%)	1.0 (2.7%)	

Primary school	88 (25.7%)	76.0 (25.3%)	12.0 (32.4%)	

Secondary school	236 (69.0%)	213.0 (71.0%)	23.0 (62.2%)	

High school	4 (1.2%)	3.0 (1.0%)	1.0 (2.7%)	

University	3 (0.9%)	3.0 (1.0%)	0.0 (0.0%)	

**After–school study time (min/day)**				0.4641

Median (IQR)	60 (60, 90)	–	–	

Mean (SD)	–	68.6 (40.1)	63.6 (36.8)	

Range	–	0.0–240.0	0.0–120.0	

**Screen–watching time (min/day)**				0.6021

Median (IQR)	30 (0, 60)	–	–	

Mean (SD)	–	44.5 (61.9)	50.0 (55.6)	

**Screen–watching distance (cm)**				0.1012

≥35 cm	229 (72.0%)	208.0 (73.2%)	21.0 (60.0%)	

<35 cm	90 (28.7%)	76.0 (26.8%)	14.0 (40.0%)	

**Exposure to daylight (hours/day)**				0.4492

≥2 hours	213 (98.0%)	191.0 (97.4%)	22.0 (100.0%)	

<2 hours	5 (2.3%)	5.0 (2.6%)	0.0 (0.0%)	


Note: Statistics presented as mean (SD), median (IQR), and n (%).

### Multivariate analysis

A multivariate logistic regression analysis was performed to identify independent factors associated with myopia in the right eye. The model included age, sex, parental myopia, after-school study time, screen time, screen-watching distance, and daylight exposure.

Only one factor emerged as statistically significant: a screen-watching distance less than 35 cm was associated with a higher risk of myopia (OR = 1.89, 95% CI = 1.02–3.47, p = 0.042). All other factors were not significantly associated with myopia status. Full results of the regression model are presented in Table 3.

**Table 3:** Multivariate logistic regression results for myopia (right eye).


FACTORS EXAMINED	ODDS RATIO (OR)	95% CONFIDENCE INTERVAL	p-VALUE

**Age (years)**	1.02	0.89–1.17	0.782

**Gender (female vs. male)**	1.14	0.61–2.12	0.684

**Parental myopia (yes vs. no)**	1.27	0.51–3.16	0.606

**After-school study time (min/day)**	1.00	0.99–1.01	0.447

**Screen time (min/day)**	1.00	0.99–1.01	0.335

**Screen distance <35 cm**	1.89	1.02–3.47	0.042

**Light exposure <2 h/day**	1.08	0.22–5.29	0.927


## Discussion

In this study, we assessed the status of myopia and its associated factors among school-aged children and adolescents in rural areas of Southern Morocco. Our findings revealed that the prevalence of myopia among participants was 11% (95% CI = 7.8–14.4%), a rate lower than that reported in most regions globally. According to Grzybowski et al. (2020), the prevalence of myopia is high in many parts of the world, including Europe (40%), North America (42%), Asia (60%), and East Asia (73%), while lower rates are observed in Africa and South America, remaining under 10% (Dong *et al.*, 2019; Grzybowski *et al.*, 2020). Thus, the prevalence observed in our study is slightly higher than the general African average but remains considerably lower than rates reported on other continents.

Similar to our findings, Pan et al. (2018) conducted an epidemiological study on first and seventh-grade primary school children in rural areas of the Republic of China. The results showed that the prevalence of myopia was 29.4% among 7th-grade students, while it was only 2.4% among 1st-grade students, with an axial length of 23.50 mm among the former compared to 23.37 mm among the latter. The authors also demonstrated that adequate adaptation of the daily time devoted to schoolwork after school led to a 15.1% reduction in the prevalence of myopia among 7th-grade students. Additionally, adjusting for time spent outdoors resulted in a 33.4% reduction in the prevalence of myopia among 7th graders.

Regarding age and school level, previous studies have consistently indicated that younger age at onset and higher educational levels are associated with a greater risk of myopia (Jones-Jordan *et al*., 2011; Hyman, 2005; Verkicharla, Kammari and Das, 2020). Children developing myopia at an earlier age are more likely to experience faster progression (Hyman, 2005; Jones-Jordan *et al*., 2011). However, in our study, no significant association was found between myopia and either age or school level.

Parental myopia was present in only 7% of participants. As reported in the literature, parental myopia is a recognized risk factor for myopia in children and adolescents due to genetic predisposition (Recko and Stahl, 2015; Xiang and Zou, 2020). Nevertheless, it is now widely acknowledged that environmental factors are predominantly responsible for the global rise in myopia prevalence (Goldschmidt and Jacobsen, 2013). In our study, given that 93.4% of the parents were non-myopic (p = 0.05), environmental factors likely played a more substantial role.

Concerning daily habits, participants reported an average of 60 minutes per day dedicated to after-school study sessions. While some studies have implicated near-work activities as a risk factor for early-onset myopia (Saw *et al.*, 2001), others found no significant association (French *et al.*, 2013; Jones-Jordan *et al*., 2011; Medina, 2021; Pan *et al.*, 2018; Xiang and Zou, 2020). Our findings are consistent with the latter, showing no significant association between after-school study time and the occurrence of myopia.

Regarding digital device use, participants reported an average of 30 minutes per day, with 72% maintaining a distance of ≥35 centimeters from the screen. The duration of screen use was not associated with myopia, but a closer screen-viewing distance was significantly associated (p = 0.0412). This observation is consistent with the literature indicating that close-up screen use constitutes a risk factor for myopia (Saxena *et al.*, 2021; Sherwin *et al.*, 2012; Xiang and Zou, 2020). The relatively short screen time and the appropriate screen-viewing distances among our participants could partly explain the low prevalence of myopia observed.

Outdoor activity and exposure to natural light were notably high among participants, with 98% reporting at least two hours of outdoor activity per day. Spending time outdoors is recognized as the most influential protective factor against myopia development (Grzybowski *et al.*, 2020; Xiang and Zou, 2020; Goldschmidt and Jacobsen, 2013; French *et al.*, 2013; Medina, 2021; Saw *et al*., 2001; Sherwin *et al.*, 2012; Saxena *et al.*, 2021). In Southern Morocco, outdoor activities are preferred over indoor or digital activities, likely due to cultural, educational, and socioeconomic factors, contributing to a behaviour that is protective against myopia.

The living environment of the participants also constitutes a protective factor. The study population originated from Zagora and Bleida, two areas characterized by a hot, dry desert climate and low urbanization (Karmaoui, 2019). Urbanization has been widely identified as a major risk factor for myopia (Dong *et al.*, 2019; Xiang and Zou, 2020). Moreover, the relatively low socioeconomic status and low population density of these areas may have contributed to the reduced prevalence of myopia, as indicated by previous studies (Dong *et al.*, 2019; Saxena *et al.*, 2021).

This study has several strengths and limitations. A major limitation is the non-representative nature of the sample, which does not allow generalization of the results to all regions of Morocco, particularly urban areas (Boutayeb, 2006). In addition, the use of a non-validated questionnaire represents a methodological limitation. Although it was not subjected to formal psychometric validation, the questionnaire was constructed based on an in-depth review of relevant literature (He *et al.*, 2015; Rose *et al*., 2008; McCrann *et al*., 2018; Xiang *et al*., 2020) and reviewed by vision experts, providing sufficient validity for exploratory field research. Nevertheless, this study addresses an important gap, as few studies have assessed the prevalence of myopia in rural Moroccan children. A major strength is the use of cycloplegic autorefraction, which is the gold standard for epidemiological studies on refractive errors and prevents the overestimation of myopia prevalence (Grzybowski *et al.*, 2020).

### Recommendations

Based on our findings, two main recommendations emerge. First, we recommend continuing epidemiological surveillance of myopia progression among children and adolescents in both rural and urban Moroccan regions. Although prevalence remains lower in rural areas, studies suggest an increasing trend even outside urban centers (Saxena *et al.*, 2021; Xiang and Zou, 2020). Future studies should systematically use cycloplegic refraction for greater accuracy.

Secondly, preventive strategies should be implemented, focusing on promoting regular ophthalmological examinations and developing health promotion programs targeting children’s visual habits. Such interventions have been shown to positively influence children’s health behaviors and outcomes (Cushing *et al.*, 2014; Cushing and Steele, 2010; Derwig, Tiberg and Hallström, 2020). Preventive messages should emphasize encouraging outdoor activities over indoor or screen-based activities, as this behavior has a positive impact on emmetropisation. Moreover, it is essential to educate children, adolescents, and their families about maintaining an appropriate distance from digital screens to help preserve eye health (Medina, 2021; Recko and Stahl, 2015)

## Conclusion

The public health problem of myopia does not spare Morocco nevertheless a minority of our participants were myopic. The two cities assessed in this study were rural with a lower overall socioeconomic status compared to larger Moroccan cities. These characteristics appear to be protective factors against myopia because children’s and adolescents’ habits are different from those living in urban areas, especially in terms of outdoor activities. Screen-watching distance appears to be the main risk factor for myopia. Actions to prevent myopia in Moroccan children and adolescents in all regions are needed as myopia.

## Competing Interests

The authors have no competing interests to declare.
